# Participant views of case finding for COPD after lung cancer screening: the C FIND COPD study

**DOI:** 10.1136/bmjresp-2025-003875

**Published:** 2026-09-15

**Authors:** Olivia R Phillips, Tricia M McKeever, Joanne Porte, Paula C A Almeida, Emma L O’Dowd, Rachael L Murray, Charlotte E Bolton

**Affiliations:** 1 NIHR Nottingham Biomedical Research Centre, Nottingham, UK; 2 Lifespan and Population Health, School of Medicine, University of Nottingham, Nottingham, UK; 3 Centre for Respiratory Research, Translational Medical Sciences, School of Medicine, University of Nottingham, Nottingham, UK; 4 Research and Innovation, Nottingham University Hospitals NHS Trust, Nottingham, UK

**Keywords:** COPD, Pulmonary Disease, Chronic Obstructive, COPD epidemiology

## Abstract

**Background:**

Early and accurate diagnosis of chronic obstructive pulmonary disease (COPD) is essential to ensure correct management and minimise future morbidity. Therefore, case finding in symptomatic at-risk individuals is highly valuable. The C FIND COPD study invited symptomatic people at risk of COPD for a COPD case-finding diagnostic assessment. These individuals had undergone a previous lung cancer screening low-dose CT. The diagnostic assessment used a mobile research unit stationed in community car parks across Nottinghamshire. This current qualitative study aimed to gather participants’ opinions about this research study and the associated lung assessment.

**Methods:**

15 participants accepted an interview using a semi-structured interview guide by an independent researcher. At the visit, seven interviewees had been given a new diagnosis of COPD and eight had no current evidence of the condition. The interviews were transcribed verbatim and analysed in NVivo (V.14) using inductive thematic analysis.

**Results:**

Across five themes, highlights included praise for the clinical research staff, the accessibility and atmosphere of the research unit, convenience of appointment and opportunity of case finding. Subsequent suboptimal communication with general practitioner surgeries was flagged as a key issue in the weeks following the assessment.

**Conclusion:**

Overall, case finding of COPD after lung cancer screening as part of the C FIND COPD study was considered a valuable assessment and experience. Interviewees were grateful for the service and aside from some practical aspects, people supported a wider roll-out.

**WHAT IS ALREADY KNOWN ON THIS TOPIC**

**WHAT THIS STUDY ADDS**

- The opportunity for COPD case finding after lung cancer screening is welcomed by participants. What is especially valued is the access provided by a community assessment. However, communication with the general practitioner surgery after the assessment is vital to ensure continued management of this chronic condition.

**HOW THIS STUDY MIGHT AFFECT RESEARCH, PRACTICE OR POLICY**

- As the National Health Service considers the opportunities for COPD case finding in those having lung cancer screening, this study offers support to the initiative.

## Introduction

 Chronic obstructive pulmonary disease (COPD) is a prevalent worldwide respiratory disease. About 1.4M people (around 1.9% of the population) have been diagnosed in the UK. A similar number of people are considered to have the condition and suffer symptoms but have no current diagnosis and thus lack treatment, meaning their symptoms go unregulated.^1 2^ COPD costs the National Health Service (NHS) £2 billion per year^3^ and if left untreated, results in more exacerbations (flare-ups), hospitalisation and premature death^2 4^ indicating that early and accurate diagnosis are imperative to optimise symptoms, prevent further deterioration and reduce the burden on the NHS.

Diagnosing COPD has its challenges. A large retrospective analysis of almost 40 000 patients in the UK found missed diagnostic opportunities in 85% of patients in the 5 years before diagnosis.^5^ A number of human factors can contribute to this delay, including a general lack of awareness of the symptoms of COPD, a misattribution of symptoms to a lack of fitness or general ageing^6^ and feelings of stigma resulting from a history of smoking—the main cause of COPD in the UK. However, case finding among symptomatic, at-risk individuals is effective and indeed has been classed as a high value intervention^7^ given the opportunity to optimise and slow deterioration to hospitalisation.

Since smoking predisposes people to both lung cancer and COPD, there have been previous endeavours to screen for COPD among people attending lung cancer screening. These approaches have usually been opportunistic spirometry alongside the CT scanning to try and detect airflow obstruction in these at-risk people. However, further diagnostic tests were then required in those with symptoms and airflow obstruction,^8 9^ resulting in multiple clinical steps and an increased workload to primary care.

In general, case finding for COPD is viewed positively by both healthcare professionals and patients, with opportunities for patients to make lifestyle changes and be more health conscious.^10^ From the perspective of clinicians, it can be resource-intensive and needs to consider what is then passed on for primary care colleagues to address after the spirometry assessment.^6 10 11^

Here, we sought research participant opinions on a proactive community-based case finding approach to COPD assessment and potential diagnosis (C FIND COPD study) after engaging with lung cancer screening. These findings are intended to complement the quantitative diagnostic findings, reported separately. In addition, we wanted to understand the subsequent events and outcomes in primary care.

## Methods

### C FIND COPD study

The C FIND COPD research study invited people to attend a diagnostic level of COPD assessment, who (1) had symptoms suggestive of COPD and (2) had previously undergone a CT scan as part of lung cancer screening in the community, and by nature therefore were at high risk of COPD. (Other at-risk, symptomatic people could also be recruited.) The assessment took place in the community on a mobile research unit (MRU), close to where people lived, a few weeks after the lung cancer screening. Importantly, the team interpreted and diagnosed the lung assessment, providing information and advice during the same visit. All results were sent to participants’ general practitioner (GP) surgery after the main clinical assessment with a view to any ongoing chronic disease management where required. It was recommended that participants contact the GP surgery a few weeks after the assessment to discuss further if not heard more.

The views of the participants were sought through a qualitative sub-study. Reporting followed the Consolidated Criteria for Reporting Qualitative Research (COREQ‐32) guidelines.

### Participants and recruitment

Participants for this qualitative study were selected using purposive sampling. They had taken part in the above C FIND COPD research assessment on the MRU 3–4 months earlier (the results of which have been written for publication separately) and had consented to being contacted in the future. This timeframe allowed the independent interviewer to seek information on the actions taken after the assessment. People were invited by telephone to participate in an interview to discuss their experience of the assessment. They were purposively selected according to whether a new COPD diagnosis had been made at the assessment but otherwise were consecutively approached from that timeframe. A patient information sheet and consent form were posted out with a prepaid envelope to return the signed consent form, if they agreed. Participants received a £10 ‘Love2Shop’ voucher in return for their time and any travel expenses incurred were reimbursed. Interviews continued until no major new themes emerged.

### Data collection

A semi-structured interview guide was developed covering the broad areas of logistical preferences, how people felt about being informed of their results, expectations of the assessment and the overall impact of the assessment on their health. The schedule was piloted in the first interview (included in analysis), and one question was added to the schedule as a result of this. During the interview, questions were tailored according to whether a COPD diagnosis had been received (see online supplemental appendix for the full interview schedule).

One-to-one interviews were conducted by ORP between November 2024 and January 2025. These were either in person at research facilities at the Nottingham Health Recovery Research Facility, City Hospital campus, Nottingham University Hospitals (NUH) Trust, or over the telephone if the participant wished. No relationships had been established between the interviewer and participant prior to the interview. Interviewees were informed that the interviewer was a public health research assistant at the University of Nottingham and part of this role was to conduct these interviews. Field notes were made after the interviews to capture and reflect on key concepts. Interviews were audio-recorded and transcribed verbatim by a NUH Trust approved external transcription company, with the participant study ID from the C FIND COPD project maintained to anonymise transcripts. There were no repeat interviews and due to timing constraints, transcripts were not returned to participants for comment.

### Data analysis

Transcripts were analysed in NVivo (V.14) using inductive thematic analysis,^12^ which allows codes and themes to be directly derived from the content of the data. One researcher (ORP) familiarised and carried out line-by-line coding of transcripts. Codes that shared a similar underlying concept were then collapsed into themes. A coding tree was created to organise and facilitate this iterative process. Regular discussions were held with CEB, RLM and TMM throughout analysis. The coding framework, theme structure and interpretations were reviewed by these researchers, who independently examined coded extracts and thematic summaries. Alternative interpretations of the data were discussed within the research team, and themes were refined through an iterative process until agreement was reached that they accurately reflected participants’ accounts.

### Patient and public involvement

The role of case finding for COPD among those having lung cancer screening was raised and highlighted at the Taskforce for Lung Health diagnostics group, which brought many patients together with clinicians (including CEB). The rationale and plans for C FIND COPD were discussed extensively with a few outpatients with COPD prior to protocol development. They were also discussed with several patient support ‘Breathe Easy’ groups across Nottingham at the planning and set-up stage.

Patients with COPD were consulted and involved in the design and set-up stages of the main study and this qualitative work. We will feed back to (1) the participants who have expressed an interest in knowing more, through a study-specific PPI meeting; (2) through further Breathe Easy groups planned over 2025/2026 and (3) through wider respiratory BRC PPI meetings we hold. In addition, we will work on an artistic interpretation of the results to share with patient support groups. This is with the direct guidance of patients who have offered input.

### Reflexive statement

This qualitative study was guided by a descriptive phenomenological perspective, with the researcher seeking to remain open to participants’ accounts and to understand their lived experiences of COPD case finding. While descriptive phenomenology encourages researchers to reflect on and bracket their preconceptions where possible, it is important to acknowledge that complete neutrality is not assumed.

The interviews were conducted by ORP, a female research assistant employed within a public health team who had experience in qualitative research but no prior clinical relationship with participants or a professional background in respiratory care and not part of the C FIND COPD team. Having this position may have reduced assumptions arising from clinical involvement; however, her public health perspective inevitably shaped the research process and interpretation of the data. Reflexivity was maintained through the use of a reflexive journal and regular discussions within the research team to consider how the researcher’s perspectives and assumptions might influence data collection, coding and interpretation.

## Results

Of 17 people approached, 15 participants agreed and gave consent to participate in an interview (one agreed to participate but did not return the consent form, and one could not commit the time). Four interviews were completed in person and 11 over the telephone. The length of interviews ranged from 20 to 50 min (mean length 33 min); telephone interviews tended to be shorter but there was no difference in the depth of information collected. Seven interviewees had been given a diagnosis of COPD through C FIND COPD and eight had no evidence of COPD at the C FIND COPD assessment. Approximately half the sample were male (n=8), all were White British, and the average age was 68 years. Participant characteristics are summarised in table 1.

**Table 1:** Demographic information of participants interviewed

N (%)	15 (100)
Gender	
Male	8 (53)
Female	7 (47)
Ethnicity	
White British	15 (100)
Age (years)	
55–59	2 (13)
60–69	7 (47)
70–74	6 (40)
Average age (years)	68
IMD quintile*	
1	3
2	3
3	3
4	4
5	2
Evidence of COPD symptoms	
Yes	7 (47)
No	8 (53)
Sites of MRU for C FIND COPD assessment (numbers represent different sites)	
1	1 (7)
2	10 (67)
3	1 (7)
4	3 (20)
Type of appointment	
Booked slot	13 (86)
Drop in	1 (7)
Can’t remember	1 (7)
Smoker at the time of appointment	
No	9 (60)
Yes	6 (40)
Has taken part in previous research?	
Yes	8 (53)
No	7 (47)
Would participate in future research?	
Yes	14 (93)
No	1 (7)

**Figure 1:**
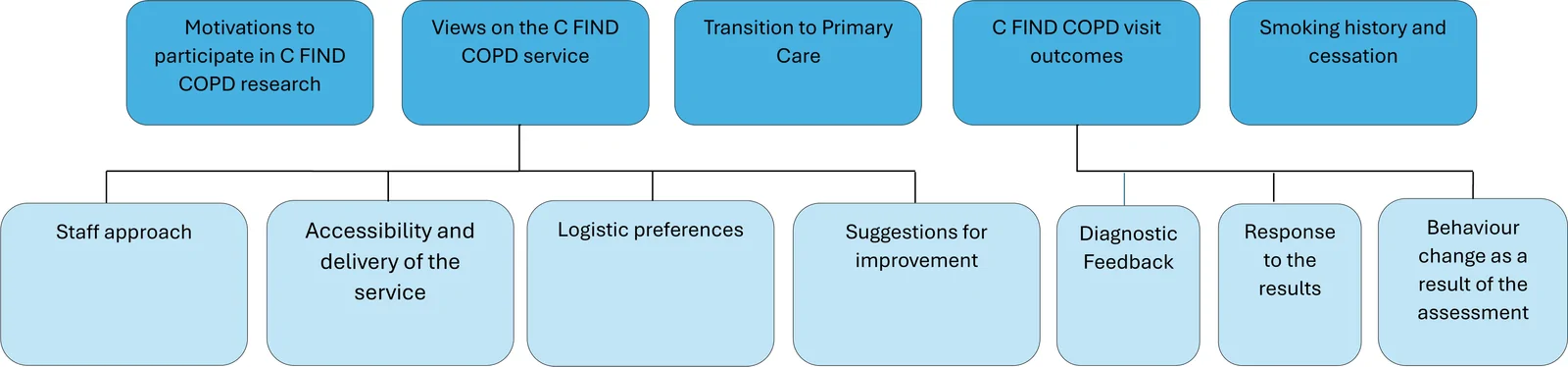
Thematic map from analysis of interviews. COPD, chronic obstructive pulmonary disease.

Analysis identified five themes and six subthemes (see figure 1). These are presented in table 2 with illustrative quotes, alongside COPD diagnosis (or not) and male (M) or female (F).

* Index of Multiple Deprivation (IMD; 1—most deprived, 5—least deprived).

COPD, chronic obstructive pulmonary disease.; MRU, mobile research unit.

**Table 2:** Themes, subthemes and supporting quotes from the participant interviews

Theme	Subtheme	Quote
Motivations to participate in C FIND COPD research		From a selfish point of view, I used to be a heavy smoker so it was nice for me to know. I mean I gave up smoking 30-odd years ago so it’s nice for me to know that my lung function is OK after being a heavy smoker for about 20 years (ID6, no COPD, F) I’m very happy being used as a guinea pig for anything because I think at my stage in life there’s not that many things you can be useful about, but being used with research is one of those things that may help future generations (ID7, no COPD)
Views on the C FIND COPD service	Staff approach	[they were] very polite. A happy face. I mean the fact that, you know if people are a bit, I don’t know, sullen or whatever, it makes you feel a bit uneasy. But the fact that they were bright and cheerful and happy and welcoming … I think it makes it so much better. It didn’t feel in any way uneasy or anything else (ID12, no COPD, M) Before the tests began, everything was again explained to me what would happen and how to breathe to get a nice result and what they were looking for and how to use the machinery … it was very clearly explained … oh yes, the girls were very knowledgeable, very knowledgeable indeed, I got lots of good advice (ID8, no COPD, M) It wasn’t hurried or, you know, it went at a nice pace and when we were testing as well, it was are you ready, you know, I wasn’t forced, see, ‘oh no we’ve got to get you done in ten minutes or so, we've got to be quicker at this’, it wasn’t that sort of atmosphere at all, it was just take your time (ID8, no COPD, M)
	Accessibility and delivery of the service	I don’t have a car of my own so I rely on public transport, but the location was in a good location to get to so there were no issue (ID8, no COPD, M) It was very informative … all the spirometry and the other tests that the young ladies did went virtually 99.9% as described in the literature. So, no, it was very plainly set out … I know sometimes it can be language that’s a bit flowery but this was very concise and very plain English I think is the phrase I’d be using (ID8, no COPD, M)
	Logistic preferences	I prefer the bus. I find it less daunting. Because you sometimes get a bit nervy, well I do, in a hospital environment. In the bus, they’re still doing the same thing, and to me it’s a nicer atmosphere (ID14, COPD, F)
	Suggestions for improvement	The only thing I would mention … somebody a bit older than me and who might be a bit frail, the steps outside the mobile unit were quite steep (ID4, COPD, F) [Referring to questions about breathlessness] … saying walking up hill, I said ‘is that a steep hill or a long hill? Some of those [questions], they weren’t precise enough for my liking if you know what I mean, but I do question things a lot (ID11, no COPD, F) It’s very hard to do that lung test thing when you think you’ve got no breath left and she’s still telling you to breathe out, which was quite difficult, but it was fine. (ID6, no COPD, F)
Transition to primary care		I did get in touch with them [the GP] and they still never got back to me on anything … I spoke to the receptionist and she said I’d get the doctor to confirm or not confirm … she looked at her monitor and says ‘well we’ve not heard anything from the people that do the testing’. And that was a month later (ID2, COPD, M)
C FIND COPD visit outcomes	Diagnostic feedback	I think they asked me that question, but I think if it were done at the same time that would be great, wouldn’t it, saves a journey don’t it (ID1, COPD, M) I don’t like being kept waiting and waiting for a letter to come and, you know, and you think the worst all the time then don’t you … my husband, he can’t cope with stress (ID9, COPD, F)
	Response to the results	I wasn’t overly surprised to be honest—I mean, I admit it, I am a smoker, I’ve been a smoker for years and years, you know, I get short of breath walking and stuff like that and going upstairs and that, so it wasn’t a total surprise (ID5, COPD, F) I was quite surprised, because I’ve always been dead fit and active, you know, chasing around, gardening, I do everything … so it’s one of those, but I haven’t smoked for, what, ten/twelve years, but I’ve still got it (ID9, COPD, F) I think knowledge is power isn’t it? And being told you’re in the, I’ve been reminded of my vulnerability … it’s a bit more information about me. Yeah, you know what you’re dealing with don’t you? You’re not sticking your head in the sand (ID15, no COPD, M) I do get breathless, so it was nice to be told that my lungs were functioning properly and so therefore I’m thinking my breathlessness is probably about stress and not some lung illness, you know, ailment or whatever, so it was very reassuring for me (ID6, no COPD, F)
	Behaviour change as a result of the assessment	If I could walk away from a main road, I do. If there’s all diesel fumes and stuff, I hold my breath till I’ve got past it, things like that. If some people seem to get a lot of smoke out, cigarettes or something, say at the bus stop, I stand away from it. I minimise all kind of smoke and obvious pollution damage to my lungs as a result of that test. (ID15, no COPD, M) We’ve [a friend and I] made a pact that we’re going to try and do that [quit] next year … it’s something I need to break (ID14, COPD, F) To be fair, my attitude changed when I went through all the heart problems, you know, obviously I don’t smoke and, like I say, you don’t drink and just keep doing swimming every day, so I’m still doing things like that (ID10, no COPD, M)
Smoking history and cessation		I am addicted to cigarettes … My life revolves round them. Yeah. It’s a pattern that I’ve got into and it’s something I need to break (ID14, COPD, F) I’m on my own at the moment … boredom is one of the biggest things—especially if you’re in the house … and then you get bored and I’d smoke because I think smoking used to, fills that gap (ID1, COPD, M) They did tell me they’d got Green Leaf and all the different things and I could be supported … I’m not interested at the moment I’m afraid. I said that I was very bad and they couldn’t persuade me to give up (ID11, no COPD, F) I was given a leaflet [on smoking cessation] and spoken to about giving up smoking. It’s another person saying “Come on, you need to give up those cigs” (ID14, COPD, F) Well, it [talking about smoking] doesn’t make you feel very good. I have tried to stop smoking, but it’s one of the most difficult things I’ve ever tried to do (ID4, COPD, F)

COPD, chronic obstructive pulmonary disease.

### Motivations to participate in C FIND COPD research

When asked why they took part in the current research, responses could be categorised into two rationales: for personal gain (ie, to get their health checked due to having a history of smoking or working in industries with dust exposure) and to give back and help others and the future generation. These were not mutually exclusive, and occasionally interviewees cited both as a reason to participate. When asked if they would participate in future research, 14/15 said yes.

### Views on the C FIND COPD service

This theme discussed interviewees’ views on a variety of aspects of the C FIND COPD assessment. Within this broad theme were four subthemes, regarding views on the staff delivering the assessment, practical aspects of the service (convenience, the MRU environment and clarity of information provided) and logistical preferences. Although mostly positive, some interviewees provided feedback on areas that could be improved.

#### Staff approach

Staff on the MRU were spoken about positively in all interviews and were one of the main contributors to the ease and sometimes enjoyment of the assessment. Specifically, they were perceived to be friendly, informative, efficient and thorough. Participants were made to feel welcomed and guided through the assessment. Staff shared their knowledge with the participants, enabling a smooth appointment where participants knew exactly what to expect at each stage. Staff were praised for being prompt; however, this efficiency never came at the expense of their patience. One interviewee commented that the staff were never in a hurry, created a relaxed atmosphere and were able to take their time. Staff ensured to capture a holistic picture of the participant’s health status, which involved conversations about smoking, breathlessness and general health.

#### Accessibility and delivery of the service

Participants expressed that the location and opening times of the MRU were convenient and that the time taken to travel there was minimal. For those where the travel time was slightly longer, they said that attending the assessment was worth it. The MRU was perceived as spacious, clean and calm, and the written material provided (in the form of leaflets about COPD, what to expect at the appointment, etc) was informative, accessible and clear to those who read them. During the interviews, certain questions aimed to identify preferences concerning how interviewees would like the service to be run. Just over half (eight interviewees) would have preferred to have their COPD assessment and lung CT scan in the same appointment, as this was viewed as less disruptive and more efficient. Three would have preferred to keep the appointments separate; four were indifferent. There was also a strong preference (12 interviewees) for the COPD assessment to take place in the MRU, rather than the hospital, due to a dislike of the hospital environment. In comparison, the MRU was deemed to be more welcoming, convenient and relaxed. Although feedback for the MRU was positive overall, there was some constructive criticism; although these minor suggestions were not a consensus and did not impact participant experience. These improvements can be divided into physical changes to the unit, which included the lack of available toilet and the steepness of the steps up to the unit; however, there was a lift available to combat this. Procedural elements were also commented on, such as the questions from the staff to assess breathlessness not being specific enough, and on one occasion, the appointment being too long. Difficulty using the spirometer was also mentioned. Each of the points of improvement was only mentioned once, by different interviewees.

### Transition to primary care

The interviewees with COPD reported that they had not received further information from their GP surgery after their C FIND COPD visit. Although participants had been encouraged to contact their GP, in some cases, participants expected the GP would contact them, leading to a delay in receiving their results. In other cases, when participants did contact the GP, there was no initial record of a new diagnosis in their records. The interviewee would inform their practice of their COPD result, who was unsure where this diagnosis had come from initially. It is possible that there is an issue in the communication pathway, meaning that the paperwork was being filed away without being coded or is not being received. This problem led to negative feelings such as frustration towards the surgery.

### C FIND COPD visit outcomes

This theme describes the various outcomes of the COPD assessment in terms of delivery of diagnostic information and initial participant response to diagnosis, which was varied across the sample: some people felt surprised by their diagnosis, others were expecting it or didn’t feel like it would change their life at all. This theme also includes findings on behaviour change as a result of engagement with C FIND COPD.

#### Diagnostic feedback

The majority (11 interviewees) preferred to be told about their result on the MRU, rather than going through the GP to find out at a later date. Finding out immediately prevented anxiety while waiting for a result. Participants also believed they may never find out if their result went to the GP first, due to perceptions that the NHS is overworked and underfunded. The positive environment provided by staff on the MRU and the building of rapport with participants, as described in the Staff approach section, was reported to help enhance the receptivity of the information provided.

#### Response to the results

A diagnosis of COPD was viewed differently between participants. Four out of seven who were diagnosed were expecting it due to years of smoking or being exposed to dust. In general, there was good awareness among interviewees of the factors that made them at risk of COPD. Two were surprised that they had COPD as they considered themselves generally fit and healthy or hadn’t smoked in years. One interviewee felt indifferent about the diagnosis and planned to continue life as normal. Depending on the diagnosis, the assessment provided either clarity or reassurance. Knowing that they had COPD was generally perceived positively as this label helped participants manage their symptoms and adapt to life with COPD. If they did not have COPD, participants felt reassured by the assessment instead.

#### Behaviour change as a result of the assessment

Occasionally, a diagnosis of COPD resulted in behavioural changes, such as avoidance of petrol/cigarette fumes or taking more rest when exercising, in an attempt to protect the lungs. Two interviewees also discussed quitting smoking or cutting down on cigarettes. However, some participants already had other health issues and therefore already had a proactive approach to their health. This indicates that it was not the new COPD diagnosis alone that encouraged them to live a healthier lifestyle, but rather it reaffirmed that they should continue doing this.

### Smoking history and cessation

Everyone who came for a COPD assessment was asked about their smoking, past or present. Six participants were currently smoking at the time of the visit, whereas nine had successfully quit. A number of reasons were reported for current smoking, including addiction, social interaction and boredom. Individuals attending an appointment said they were expecting to have a conversation about smoking with a member of staff as the appointment concerned lung health. Those who smoked at the time were offered support for smoking cessation. Although this was positively received by some, not everyone was interested in giving up. For one interviewee who was a current smoker, the conversation had a negative emotional effect.

## Discussion

This qualitative study provides an in-depth review of the participant experience of a COPD case finding research assessment following lung cancer screening, on board an MRU, located in communal areas close to where the invited people lived. Importantly, it captured the perspective of those with and without a new diagnosis of COPD at the time of the assessment and their experience afterwards.

The experiences of this post-lung cancer screening COPD case finding assessment mirror the wider COPD case finding literature which has previously been mainly primary care based. Most people believe that proactive case-finding is valuable and that early detection is important.^13^ There is motivation to attend case finding due to a concern for, or interest in, their own health but also to help others and to help research.^10 13^ In the literature, patients who had proven airflow obstruction were reassured by the clarity the assessment provided as they could now attribute their symptoms to COPD, which they then knew how to manage.^11 13^ Patients whose spirometry was normal felt reassured that they did not have to worry about COPD.^13^ Throughout the literature and this study, staff approachability, professionalism and knowledge have been key to the patient experience.^10^ This is important as patient–practitioner relationships are a determinant of patient outcomes and of the acceptability of information provided.^14 15^

Previous research has found that some patients express concern about the emotional impact of a potential diagnosis and reported that clinical information was occasionally too detailed, complex or not tailored to their needs.^10^ These findings highlight the importance of clear, personalised communication, knowledgeable staff and sensitive delivery of results to support patient engagement in COPD case finding initiatives. Although previous research has found COPD diagnoses to lead to feelings of stigmatisation,^10 13 16^ this was not mentioned by the interviewees in the current study. However, this may be because no question directly asked about this aspect of COPD, but rather their experiences on the MRU. When particularly considering feelings of smoking-related stigma, the fact that this was not reported by participants in the current study is potentially a positive reflection of the community-based mobile unit which offered a less medicalised setting with staff who were perceived to be friendly and welcoming, building rapport with participants and taking a holistic approach to their health and well-being. Further research is warranted in this area to fully optimise the benefits that could be gained from a wider rollout of the C FIND COPD approach discussed here.

Case finding for COPD can also result in improved health behaviours, such as people being more health conscious, losing weight and better protecting their lungs. Our findings echo previous literature that report that many patients attempt to stop/reduce smoking after their assessment and avoid dust/fumes,^11^ although not all.^10^ Given the high levels of nicotine dependence and the complex social circumstances often faced by individuals attending COPD case finding screenings, it is essential that smoking cessation support is routinely offered as part of this process. These individuals may have longstanding patterns of tobacco use linked to socioeconomic disadvantage, mental health challenges or limited access to healthcare. Providing tailored cessation interventions at the point of screening addresses a key modifiable risk factor for COPD progression and offers support in an opportune moment of potential receptiveness and need.^17^

In C FIND COPD, the apparent post-assessment miscommunication with primary care is not a new issue but is a vital hurdle to overcome.^13^ The C FIND COPD study discussion by a healthcare professional after the spirometry covered information on diagnosis and advice and information on COPD (where appropriate), symptoms and smoking cessation. However, a good transition of information and care to primary care is essential for optimal longer-term chronic disease management and considering pharmacological therapy. Access limitations to primary care have previously been reported as a barrier to attending COPD assessments, with patients citing difficulty booking GP appointments, a lack of convenience and perceived GP time constraints.^8 13^ On the back of this research, we are systematically reviewing this transition and primary care documentation more comprehensively to try and understand factors contributing to any barriers or delays experienced. These could include communication across and between services, practical components such as data processing times and patient understanding or expectation of follow-up processes and further research should investigate these factors.

The current study supports the acceptability of this approach to case finding in COPD after LCS and welcomes the experience and approach of trained personnel, not just in the spirometry procedure but the knowledge and information provided after.

### Strengths and limitations

One of the main strengths of this evaluation was the use of semi-structured interviews by an independent interviewer. Although there were a set of predetermined questions, the flexibility of this technique enabled the interviewer to diverge from the interview schedule and follow, deepen and reveal new thoughts and perspectives important to the interviewee. This evaluation also identified areas for improvement in the study, mainly the suboptimal communication between the C FIND team and primary care services. Identifying issues can work to strengthen the pathway and benefit the service for people to use in the future.

This study has drawbacks, namely that 10 out of 15 interviewees had their assessment at the same community car park and therefore they were likely registered across a small pool of GP surgeries. All participants interviewed were White, and although this demographic makes up 66% of the Nottingham population (in 2021)^18^ and the vast majority of those taking part in the wider C FIND COPD study, it does not allow for wider social and cultural views. However, even with greater representation, the views expressed may not represent those people who did not accept the invite for C FIND COPD assessment originally. This is an important limitation from an implementation perspective, as it captures the views of those already engaged and motivated with healthcare; it does not capture the views of those who do not and thus are likely at higher risk of disease; ethical approval within the current study was not in place to allow contact with those declining engagement with the C FIND COPD assessment. However, a number of practical considerations have been raised that warrant consideration if a COPD case finding was rolled out. Further, we only invited participants who had previously expressed interest in taking part in future research; however, this was over 96% of the C FIND COPD recruits and only two invitees declined or were unable to attend. The delay from assessment to interview (3–4 months) was intentional in order to capture participant experience, therapies and behaviour change after the assessment, particularly those with a new diagnosis or current smoker but may have led to issues in recall bias, most notably with regard to their feelings about the assessment process and their results. Finally, only one researcher (ORP) conducted the qualitative analysis, which introduces a level of subjectivity to the interpretation of the results. However, she was independent of the clinical team and the original setup of the study and analysis was discussed with other members of the team.

## Conclusion

Overall, the case finding of COPD after lung cancer screening, undertaken in a community-based unit in this research project, was classed as a valuable experience by those who attended. Attendees were grateful for the community-based service, which provided reassurance, clarity and an opportunity they might not have had otherwise. There remains a need to ensure effective communication with primary care to ensure effective ongoing management of disease diagnosis.

## Supplementary material

10.1136/bmjresp-2025-003875 online supplemental file 1

## Data Availability

Data are available upon reasonable request.
