# Supplementary material for: Participant views of case finding for COPD after lung cancer screening: the C FIND COPD study
Source: BMJ Open Respir Res. 2026 Sep 15;13(1):e003875. doi: 10.1136/bmjresp-2025-003875 (PMC13583836; doi:10.1136/bmjresp-2025-003875)
Supplement: Supplementary file 1 [file bmjresp-13-1-s001.docx]

**Participant Views of Case Finding for COPD after Lung Cancer Screening: The C FIND COPD Study**

Phillips OR^1,2^, Tricia M Mckeever^1,3^, Porte J^1,3^, Almeida PCA^1,4^, O'Dowd EL^1,3,5^, Murray RL^1,2,*^, Bolton CE^1,3,5,*^

**Interview schedule**

**IF INTERVIEWEE WENT FOR THE SPIROMETRY**

- Do you recall which site you went to for blowing / breathing tests? When – month
  - Clifton Morrisons
  - Hyson Green Asda
  - Bulwell Morrisons
  - Buzz Bingo top Valley
  - Strelley Social Club
  - Lidl Wollaton
- Did you get a letter asking you to take part or did you see the unit and decide to go on board?
  - If got a letter, did the letter and information sheets provide right level of information?
  - Was there too much information/did you want to know more?
- Thinking about your visit to the unit
  - What was location like – at x car park? How easy was it to get to?
  - What did you think of the unit in general?
    - Were you made to feel welcome? Did you feel comfortable?
    - Bus vs hospital environment *(question added after pilot interview)*
  - When you got the invite letter, did you take up the offer - Did you request a pre-booked time to attend or did you choose one of the days where you could drop in?
    - Did you prefer your option/would you have preferred the alternative?
    - Did you find the opening times convenient?

**IF THE INTERVIEWEE HAD THE CT SCAN**

- Would you have preferred to have breathing tests at same time as lung scan or come back at a different time?
  - was it too much fuss coming back a 2^nd^ time
  - Probe further about preference either way

**The visit / outcomes**

At the breathing test visit, we aim to tell the people the results of the clinical tests including the blowing test there and then

- Did you get told your breathing test was normal, or you needed further discussion with the surgery?
  - How did you feel about being told what you were told?

**IF ABNORMAL SPIROMETRY**

- How did you feel being told your results?
- Was the timing right or did you find it too much?
- The alternative would be we forward the results onto your GP surgery for the team there to discuss the results with you. Do you think that would be better?
- Do you recall having a conversation about your test findings with a member of the team?
  - Was this helpful
  - Did your questions get answered?
- Did the team give you information to take away?
  - What did you think of the leaflets given to you? Were they useful? Did they answer any questions you had? Have you used them?
- If you were recommended to go to the GP surgery to discuss further – has that happened / how useful has it been? Did the GP start any new treatments?

**IF SPIROMETRY NORMAL BUT INTERVIEWEE EXPERIENCES BREATHLESNESS**

- How did you feel being told your results?
- Was the timing right?
- The alternative would be we forward the results onto your GP surgery for the team there to discuss the results with you. Do you think that would be better?
- Do you recall having a conversation about your breathlessness and general health with a member of the team?
  - Was this helpful
  - Did your questions get answered?
- Did the team give you information to take away?
  - What did you think of the leaflets given to you? Were they useful? Did they answer any questions you had? Have you used them?
- If you were recommended to go to the GP surgery to discuss further – has that happened / how useful has it been? Did the GP start any new treatments?

**IF SPIROMETRY NORMAL / NO SYMPTOMS**

- How did you feel being told your results?
- Do you recall having a conversation about your general health with a member of the team?
  - Was this helpful
  - If you had any questions did they get answered?

**ALL WHO ATTENDED THE VISIT**

- If you were a smoker at the time you attended for the breathing test, did anyone talk to you about your smoking?
- If yes, how did this make you feel?
  - Were you expecting it? Did you feel it was appropriate timing?
- Were you offered advice and support to stop (include leaflet prompt)?
  - What were you offered? Did you find it helpful? Have you cut down or quit?
- If no, were you expecting to have a conversation about your smoking? Do you wish you had?
- Do you feel that the visit has been good for your health?
  - In what way? (physical activity, vaccine uptake, stopping smoking etc prompt)
- Have you ever taken part in research before or was this the first research project?
- Would you consider in the future?
- Is there anything else you would like to add?
  - If not, thanks and close interview
